# Baseline spatial distribution of malaria prior to an elimination programme in Vanuatu

**DOI:** 10.1186/1475-2875-9-150

**Published:** 2010-06-02

**Authors:** Heidi Reid, Andrew Vallely, George Taleo, Andrew J Tatem, Gerard Kelly, Ian Riley, Ivor Harris, Iata Henri, Sam Iamaher, Archie CA Clements

**Affiliations:** 1 Pacific Malaria Initiative Support Centre (PacMISC), Australian Centre for International and Tropical Health (ACITH), School of Population Health, University of Queensland, Queensland, Australia; 2 National Vector Borne Disease Control Program (VBDCP), Ministry of Health, Port Vila, Vanuatu; 3 Australian Army Malaria Research Institute, Department of Defence, Government of Australia, Queensland, Australia; 4 Emerging Pathogens Institute and Department of Geography, University of Florida, Gainesville, USA; 5 Australian Centre for Tropical and International Health, Queensland Institute of Medical Research, Brisbane, Queensland, Australia

## Abstract

**Background:**

The Ministry of Health in the Republic of Vanuatu has implemented a malaria elimination programme in Tafea Province, the most southern and eastern limit of malaria transmission in the South West Pacific. Tafea Province is comprised of five islands with malaria elimination achieved on one of these islands (Aneityum) in 1998. The current study aimed to establish the baseline distribution of malaria on the most malarious of the province's islands, Tanna Island, to guide the implementation of elimination activities.

**Methods:**

A parasitological survey was conducted in Tafea Province in 2008. On Tanna Island there were 4,716 participants from 220 villages, geo-referenced using a global position system. Spatial autocorrelation in observed prevalence values was assessed using a semivariogram. Backwards step-wise regression analysis was conducted to determine the inclusion of environmental and climatic variables into a prediction model. The Bayesian geostatistical logistic regression model was used to predict malaria risk, and associated uncertainty across the island.

**Results:**

Overall, prevalence on Tanna was 1.0% for *Plasmodium falciparum *(accounting for 32% of infections) and 2.2% for *Plasmodium vivax *(accounting for 68% of infections). Regression analysis showed significant association with elevation and distance to coastline for *P. vivax *and *P. falciparum*, but no significant association with NDVI or TIR. Colinearity was observed between elevation and distance to coastline with the later variable included in the final Bayesian geostatistical model for *P. vivax *and the former included in the final model for *P. falciparum*. Model validation statistics revealed that the final Bayesian geostatistical model had good predictive ability.

**Conclusion:**

Malaria in Tanna Island, Vanuatu, has a focal and predominantly coastal distribution. As Vanuatu refines its elimination strategy, malaria risk maps represent an invaluable resource in the strategic planning of all levels of malaria interventions for the island.

## Background

**Figure 1:**
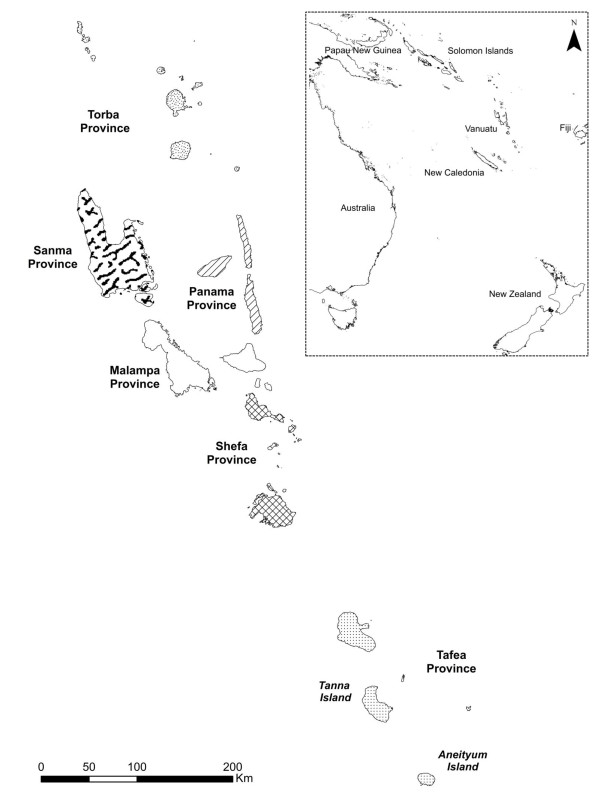
**Map of Vanuatu showing the location of Tafea Province within the country and the location of Vanuatu with respect to neighbouring countries in the Western Pacific region (inset)**.

In recent years, the momentum behind malaria elimination has gathered speed with thirty-nine countries across the world now making progress toward malaria elimination. One of the key strategies is to shrink the global malaria map from the endemic margins inward [1]. While some nations are committed to nationwide elimination, others are pursuing spatially progressive elimination within their borders. With support from international donors, the Ministry of Health in the Republic of Vanuatu has started to implement a malaria elimination programme in Tafea Province which is comprised of five islands, Fatuna, Aneityum, Erromango, Aniwa and Tanna (Figure 1). Interrupted malaria transmission has already been achieved on the island of Aneityum through the use of approaches such as mass drug administration and insecticide treated bed nets, and with the enthusiastic support of the local population [2]. Tafea Province represents the most southern and eastern limit of malaria in the South West Pacific and thus a strategic starting point for elimination activities.

While the optimal strategy for elimination is being debated, possible distinctions between control and elimination efforts are the geographical scale and intensity of key interventions [3]. During the control phase, interventions tend to be widely applied to the target areas, often with similar strategies between zones of varying endemicity. As the prevalence declines remaining transmission is increasingly restricted to specific geographical foci and more precision in the application of interventions is needed, with more intense targeting of resources to identify and eliminate the last remaining sites of transmission and/or resistance [4]. An effective elimination campaign must be capable of identifying these foci. Mapping offers great potential and the greatest need for malaria maps is at the periphery of stable, endemic areas where there is often less empirical information regarding risks and intensity of infection [5].

The advancement of geographical information systems (GIS) and spatial statistics has greatly improved our understanding of malaria dynamics, including its dependence on ecological factors [5-12]. More recently, Bayesian geostatistics has been embraced for disease mapping with the advantage that both environmental covariates and spatial autocorrelation are able to be estimated simultaneously and full posterior distributions produced, which can be used to quantify uncertainties in parameters of interest (e.g. predicted prevalence of infection)[13]. Spatial prediction models have been used to produce malaria risk maps at national [11,14-19] sub-continental [20-22] and global scales [23,24].

The aim of this present study was to produce accurate, validated predicted prevalence maps for *P. vivax *and *P. falciparum *on Tanna Island, Vanuatu. Additionally, it is envisaged that the maps be used to spatially define an implementation strategy for malaria elimination in Tanna Island. The applicability of the methods and the implications of the results are discussed in the context of malaria elimination strategies, which are beginning to take shape.

## Methods

### Survey data

Data pertaining specifically to Tanna Island (N = 4763) were extracted from the results of a parasitological survey conducted in Tafea Province in 2008 by the National Vector Borne Disease Control Program (VBDCP), Vanuatu, in collaboration with the Pacific Malaria Initiative Support Centre (PacMISC), a Brisbane-based consortium consisting of the School of Population Health at the University of Queensland; the Australian Army Malaria Institute (AMI); and the Queensland Institute of Medical Research (QIMR)[25]. Within Tanna, the school-based survey covered all 80 schools on the island. Blood samples were collected from children between two and 12 years of age by finger prick using a lancet and the samples were examined microscopically for malaria parasites. Dried blood spot specimens were also collected and transported to the AMI in Brisbane for analysis by polymerase chain reaction (PCR), considered the gold standard for malaria diagnosis, the results of which were used to develop the spatial models presented in this report.

Each child was interviewed to collect information such as school [N = 80], home village [N = 233] and village in which they usually sleep ('sleep' village, N = 233). Village coordinates were not taken at the time of the survey but school, home and sleep villages were later matched to a government list provided by the Vanuatu Ministry of Lands from a 1999 census. To most accurately match infection to place of transmission, sleep village was used as the geographical reference because this was considered a more accurate representation of exposure sites than home or school village. For sleep villages not able to be geo-referenced, the home and then school village were used as the geographical reference. If villages were not included in the census list (4% of children), the local malaria control officer on Tanna Island was consulted to determine the closest listed village to the sleep village and this was chosen as the geographical reference. A total of 4,716 children (99%) could be geo-located in this manner to a total of 220 'sleep', 'home' or 'school' villages.

**Figure 2:**
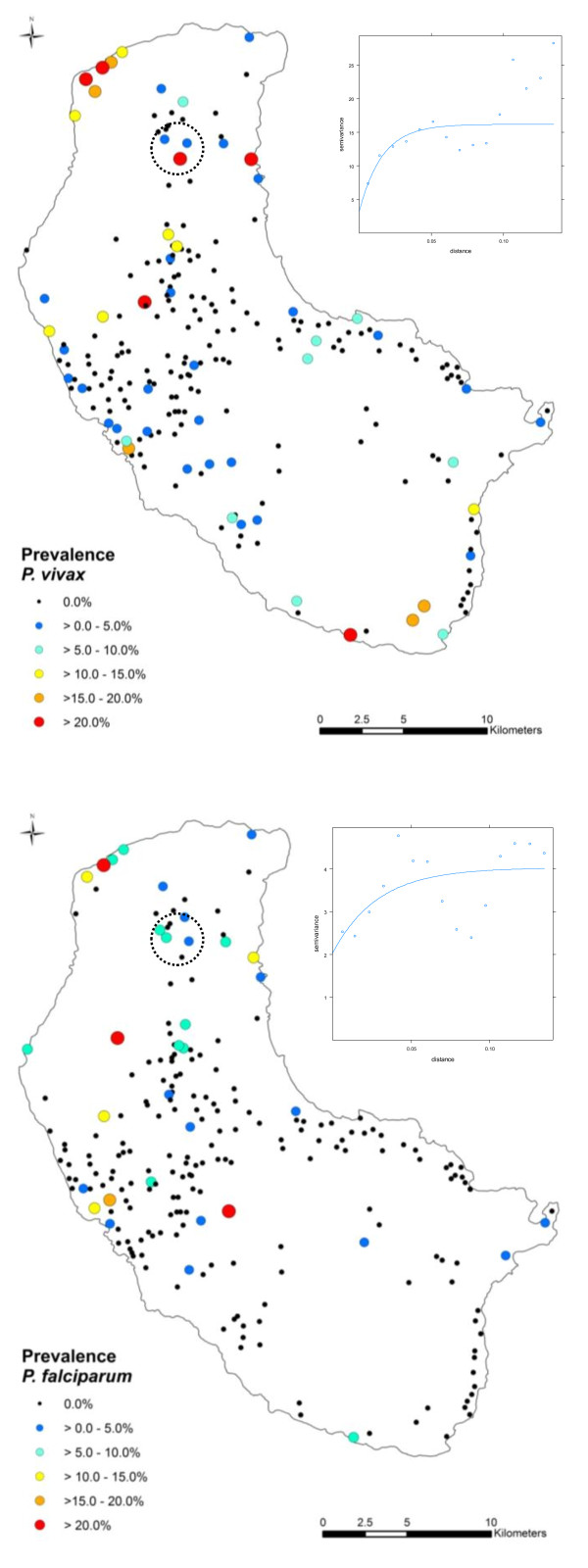
**Geographic distribution of *P. vivax *(top) and *P. falciparum *(bottom) prevalence on Tanna Island, Vanuatu based on PCR results from the 2008 parasitological survey and their associated variograms displaying spatial autocorrelation over a similar range of 0.05 decimal degrees (corresponding to approximately 5 km)**.

Four villages in the Green Hill area in the north of the island (circled in Figure 2) showed an unexpectedly high proportion of malaria-positive individuals given their inland location. As this finding was hypothesized to be an artefact of the post-survey geo-referencing method, these four villages were removed during a secondary analysis to assess their impact on the significance of environmental variables.

### Analysis of spatial structure

Statistical analyses of spatial structure in the prevalence data were done in R version 2.9.0 (The R foundation for statistical computing) using the GeoR package. For these analyses the sample variogram was defined as:

Where *p*(*x*_*i*_) represents a value of prevalence observed at location *x*_*i *_and *p*(*x*_*i *_-*h*) represents a second observation at a distance *h *from the first. By discretising lags into a series of bins of width *b*, such that each value of *h *actually represents a distance interval *h *± *1/2b*, semi-variances are computed as the mean semi-variance amongst the set of *n(h) *pairs of observations separated be distances within that interval [24].

### Assembling and testing ecological and climate variables

Images from Landsat Enhanced Thematic Mapper (ETM) were downloaded from the United States Geological Survey (USGS) Earth Resources Observation and Science Center [26]. These images were processed to develop estimates of normalized difference vegetation index (NDVI) [27] representing the amount of vegetation per 30 metre spatial resolution pixel and Thermal Infra-Red (TIR) as a surrogate for surface temperature at 60 m spatial resolution.

Gridded elevation data at 90 m spatial resolution were obtained from the Shuttle Radar Topography Mission digital elevation dataset, processed and made available at the Consultative Group on International Agricultural Research Consortium for Spatial information [28]. Finally distance to coast was calculated using the spatial analyst extension of the GIS software ArcView version 9.3 (ESRI, Redlands, CA). The same software was used to extract NDVI, TIR and elevation for the 220 village locations.

Colinearity between each pair of environmental variables (NDVI, TIR, elevation and distance to coastline) was assessed in Stata/SE Version 10 (Stata Corporation, College Station, TX, USA) statistical software package with 0.9 defined as the cut-off. If co-linearity was observed separate models incorporating the different variables would be constructed. Backwards step-wise regression analysis was conducted on the remaining variables to determine their inclusion into the final spatial prediction model. Those variables with a *p*-value < 0.1 were retained.

### Bayesian geo-statistical model

**Figure 3:**
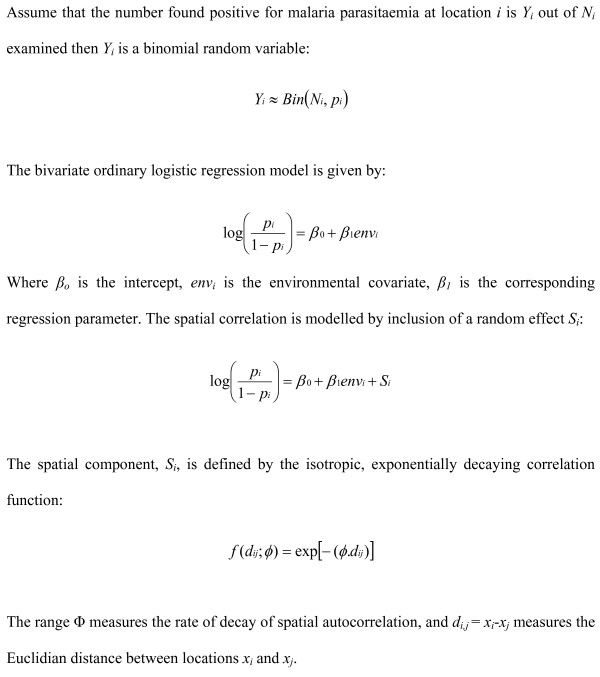
**Spatial prediction model based on the principles of model-based geostatistics**.

A spatial prediction model (Figure 3) was constructed based on the principle of model-based geostatistics [29] in the Bayesian statistical software WinBUGS version 14.1 (MRC Biostatistics Unit, Cambridge, UK). The model comprises two components: a deterministic component consisting of village-level fixed effects; and a stochastic component consisting of an isotropic, stationary autocorrelation function describing village-level spatial variation (i.e. a geostatistical random effect). To predict the prevalence at unsampled locations, a grid was generated of 1958 prediction locations with a spacing of 0.01 decimal degrees (approximately 1 km), covering the island. Using the in-built *spatial.unipred *function in WinBUGS, the geostatistical random effect was interpolated to all prediction locations and predicted prevalence was calculated by adding the random effect to the sum of the products of the coefficients for the covariates and the values of the covariates at each prediction location.

### Model validation

Validation of predicted prevalence was undertaken by partitioning the data into four random subsets, running the model using three of the four subsets and validating the model with the remaining subset. Four separate models were run, each with a different subset excluded for validation. The accuracy of the prediction was determined in terms of area under curve (AUC) of the receiver-operating characteristic (ROC), with observed prevalence, dichotomised at 0 and ≥0, taken as the comparator. This gave an indication of the ability of the model to discriminate between areas where transmission did and did not occur. As a general rule, an AUC between 0.5 and 0.7 indicates a poor discriminative capacity; 0.7-0.9 indicate a reasonable capacity; and >0.9 indicate a very good capacity.

## Results

The overall prevalence of *P. falciparum *and *P. vivax *infection was 1.0% (95% CI: 0.79-1.21%) and 2.2% (95% CI: 1.06-3.34%) respectively [25] with the spatial distribution broadly similar between the species (Figure 2).

Semivariograms (Figure 2) revealed spatial autocorrelation was a feature of raw prevalence of *P. vivax *and *P. falciparum*. Colinearity of 0.91 (measured using Pearson's correlation coefficient) was observed between elevation and proximity to coastline. NDVI and TIR were not statistically significant predictors and thus were not included in the final models. Exclusion of the four aforementioned villages did not affect the significance of environmental variables and thus a separate model was not deemed necessary.

**Figure 4:**
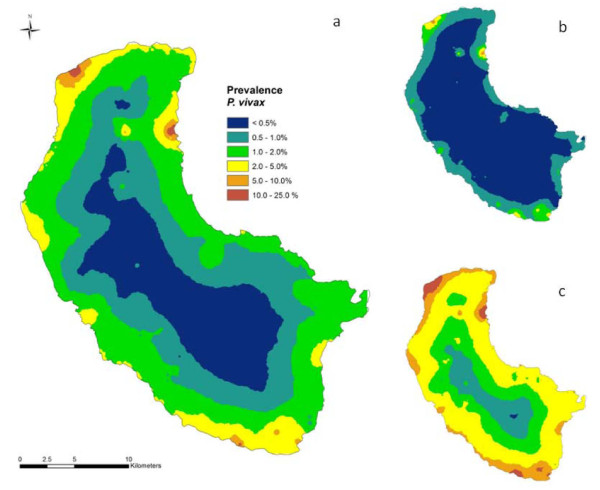
**a) Median predicted spatial distribution of *P. vivax *prevalence on Tanna Island, Vanuatu in 2008 at approximately 1 km^2 ^resolution, b) lower 25% predicted prevalence, and c) upper 75% predicted prevalence**.

**Figure 5:**
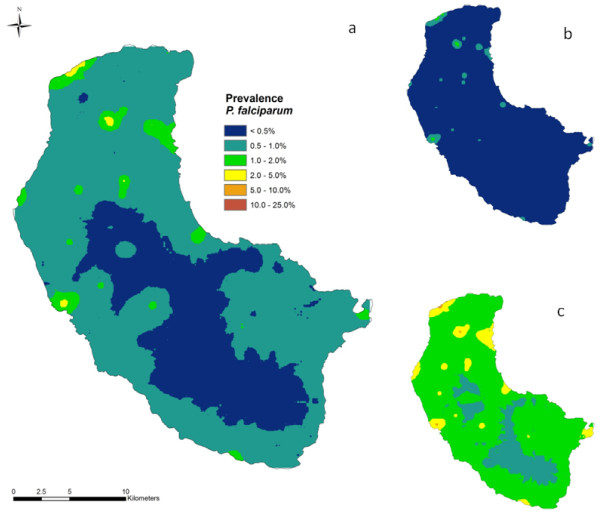
**a) Median predicted spatial distribution of *P. falciparum *prevalence on Tanna Island, Vanuatu in 2008 at approximately 1 km^2 ^resolution, b) lower 25% predicted prevalence, and c) upper 75% predicted prevalence**.

Tables 1 and 2 present the results of the Bayesian geostatistical models for *P. vivax *and *P. falciparum*. For *P. vivax*, the model with distance to coastline as the fixed effect gave the best fit with better predictive ability and lower mean error and mean square errors values than the model with elevation as the fixed effect. For *P. falciparum*, the model with elevation as fixed effect gave the best fit. On the basis of the models and validation it was possible to predict the distributions of *P. vivax *and *P. falciparum *risk across the island (Figure 4 and Figure 5 respectively). The strong relationship between proximity to coastline and *P. vivax *gave a smooth risk map with endemicity classes following the contour of the coastline. The risk maps for *P. vivax *and *P. falciparum *include the upper and lower Bayesian credible limits and show the extent of uncertainty in predicted malaria risk in 2008.

**Table 1:** Results of Bayesian geostatistical models to predict prevalence of *P. vivax and P. falciparum *for Tanna Island, 2008.

	Coefficient, posterior mean	Odds ratio, posterior mean (95% Bayes credible intervals^#^)	DIC
***Model of P. vivax with distance to coastline fixed effect***			

*α *(intercept)	^-^4.680 (^-^5.317- ^-^4.137)		

Distance from coastline (OR per 1 km)	^-^0.690 (^-^1.151- ^-^0.242)	0.730 (0.591-0.895)	

*Φ *(rate of decay of spatial correlation)*	251.5(51.26-569.4)		

*σ*^2 ^(variance of geostatistical random effect)**	0.214 (0.056 - 0.624)		

*DIC*			306.9

***Model of P. vivax with elevation fixed effect***			

*α *(intercept)	^-^4.611 (-5.327 - ^-^3.931)		

Elevation (OR per 100 m)	^-^0.547 (^-^0.992- 0.115)	0.654 (0.468, 0.917)	

*Φ *(rate of decay of spatial correlation)*	167.8 (33.71, 461.2)		

*σ*^2 ^(variance of geostatistical random effect)**	2.471 (1.271, 4.304)		

*DIC*			308.9

***Model of P. falciparum with distance to coastline fixed effect***			

*α*	^-^5.238 (^-^6.027 - ^-^4.625)		

Distance from coastline (OR per 1 km)	^-^0.101 (^-^0.534 - 0.334)	0.955 (0.783, 1.165)	

*Φ*	289.5 (51.61 - 575.4)		

*σ*^2^	0.584 (0.057 - 4.713)		

*DIC*			219.5

***Model of P. falciparum with elevation fixed effect***			

*α*	^-^5.129 (-5.976 - ^-^4.416)		

Elevation (OR per 100 m)	^-^0.207 ^(-^0.673 - 0.146)	0.864 (0.603, 1.149)	

*Φ*	238.2 (53.28 - 478.9)		

*σ*^2^	1.753 (0.4521 - 4.079)		

*DIC*			218.9

* The unit is change in spatial autocorrelation per decimal degree. A lower Φ indicates that spatial correlation occurs over longer distances (i.e. spatial clusters are larger).

** A higher variance indicates a greater tendency toward spatial clustering.

# Bayes credible intervals can be interpreted as having a similar meaning to confidence intervals used in frequentist statistics.

**Table 2:** Summary of validation statistics for the geostatistical models described in Table 1.

Model	AUC	Mean Error^# ^(% prevalence)	Mean Absolute Error* (% prevalence)
***Model of P. vivax with distance to coastline fixed effect***	0.867	5.07	1.30

***Model of P. vivax with elevation fixed effect***	0.857	5.46	1.33

***Model of P. falciparum with distance to coastline fixed effect***	0.821	0.39	0.55

***Model of P. falciparum with elevation fixed effect***	0.856	0.34	0.50

AUC between 0.5 and 0.7 indicates a poor discriminative capacity; 0.7-0.9 indicate a reasonable capacity; and >0.9 indicate a very good capacity.

# Mean error is a measure of the bias of predictions (the overall tendency to over or under predict).

* Mean absolute error is a measure of overall precision (the average magnitude of error in individual predictions).

## Discussion

The Bayesian prediction models clearly show that transmission is not homogeneous, with malaria risk displaying a predominately coastal distribution, concentrated within well-delimited foci or 'hot-spots'. While the presence of malaria foci on the island is supported by anecdotal evidence the presence of an inland 'hot-spot' in the Green Hill area is contentious. An entomological survey carried out on the island concurrently with the parasitological survey did not reveal any vector breeding sites in the Green Hill area and concluded that transmission is restricted to within 2 km of the coast. It appears that the Green Hill area, which is approximately 5 km from the coast, does not support the local vector and that malaria infections were contracted whilst inhabitants visited coastal areas. The Green Hill area focus requires further investigation, particularly with respect to patterns of mobility for work and recreation. In addition, a better understanding of the transmission patterns could be obtained from surveying *Anopheles farauti *breeding sites on the island during the dry season.

The risk maps have been used to define zones within which interventions are scaled and planned according to malaria transmission intensity. This will involve indoor residual spraying (IRS) on the coastal fringe but not the hinterland/middle bush area. Additionally the maps provide the base for the design of the surveillance strategy and will be fully implemented by targeted surveys which will in turn inform modifications in local implementation policies such as inclusion of a particular inland hotspot for IRS.

Whilst the maps of the upper and lower Bayesian credible intervals are important for scientific honesty (i.e. highlighting areas where the predictions are imprecise) they are also a useful tool for priority setting and thus aiding in the sound and rational deployment of interventions. The maps showing the lower Bayesian credible intervals are particularly useful in highlighting those locations in which there is high certainty of above-average malaria prevalence. Active surveillance within high-prevalence foci is a cornerstone for success in interrupting malaria transmission [30]. The upper Bayesian credible intervals are useful in providing an indication of the maximum extent of malaria transmission or risk.

At the community level the risk maps have been highly successful in planning meetings. The importance of grassroots-level involvement and local ownership of the elimination goal is well recognized [2]. The island has had a long history of malaria control and such maps are an effective way for local citizens to visualize and understand the strategy for the elimination programme.

Initial statistics analysis of environmental covariates revealed no association between malaria risk and NDVI or TIR. At the small geographical scale of our study (550 km^2^), NDVI and TIR are relatively homogeneous and thus not indicative of the presence or absence of malaria. NDVI has been positively associated with malaria prevalence throughout Africa [8,18,22,31,32] and the Middle East [14], but these studies covered wide geographical areas exhibiting substantial variations in climate. The relatively small size of the study area meant that the commonly used source of vegetation cover, Globcover [33], was not available at a small enough resolution to usefully define vector habitats of *An. farauti*.

The present study found *P. vivax *risk to be associated with proximity to the coastline, which has been identified by others as a desirable *An. farauti *habitat [34,35]. It is believed the relatively small number of *P. falciparum *cases detected on the island were not sufficient to yield conclusive results concerning associations with environmental variables. Thus despite both *P. vivax *and *P. falciparum *being supported by the same vector species slightly different relationships with environmental variables were observed.

As the need to define the limits of malaria distribution and to predict the distribution within these limits increases, the utility of ecological and climatic variables in aiding this endeavour have been questioned. While an issue that cannot be resolved within this brief discussion suffice to say that as vectors' biological niches continue to be determined by ecological and climatic factors (along with human related socio-economic factors) understanding and identifying these variables will continue to be important. However, at differing spatial scales and for different vector species the significance of ecological and climatic variables will vary. For example, increases in altitude are commonly associated with cooler temperatures and thus a less suitable environment for malaria transmission for much of Africa [36-38], while in the forested hills of South-East Asia increased altitude has been found to be associated with increased forest cover and thus a more suitable transmission environment for the vector species in this ecological niche [39,40]. Looking at smaller spatial scales, variables such distance to local water bodies and a households' location with respect to the village periphery become important determinants of malaria risk [10,12].

Accurate data on distribution of population across the island was not available at the time of this study but has been identified as a priority for the malaria elimination programme. However, the population is described to be is spread over the entire island with an inland plateau region more densely populated [25]. Once more accurate population data is available a better understanding of disease burden is possible.

While these maps will serve as baseline maps for the elimination programme such intensive surveying (i.e. 76% of all children aged 2-12 years [25]) will not be necessary to update the maps. As surveillance operations become more streamlined the routine data collected from peripheral aid posts can be used. Additionally, the data collected throughout the year can be adjusted for seasonality [24] to provide more accurate estimates of average annual prevalence.

The current study does come with some limitations, particularly the geo-referencing method, which was applied retrospectively using independently sourced coordinates (rather than during the survey using a global positioning system). Additionally, the cross-sectional survey design meant that prevalence data were obtained at a single time point and thus the data only represent a snap-shot of malaria risk, which is known to have important temporal dynamics. Despite these limitations, as data are updated with the input from surveillance operations, the spatial predictions presented here represent the baseline of a dynamic, and ideally shrinking, malaria risk map for Tanna Island.

## Competing interests

The authors declare that they have no competing interests.

## Authors' contributions

PacMISC, AMI and the VBDCP conceived the study and participated in its design and data collection. AMI assembled the bulk of the incidence data. AC and HR devised and implemented the analytical methods. AT provided processed satellite images for analysis. HR wrote the first draft of the manuscript. All authors participated in the interpretation of results and in the writing and editing of the manuscript.
